# Intergenerational family support and older adult frailty: findings from four prospective cohort studies

**DOI:** 10.1016/j.ssmph.2026.101934

**Published:** 2026-05-30

**Authors:** Wenze Tian, Xiaoyu Wang, Gang Hu, Zheng-Hui Zhao

**Affiliations:** a School of Social Development, Tianjin University of Technology, Tianjin, 300384, China; b School of Public Administration, South China Agricultural University, Guangzhou, 510642, China; c School of Health Management, Xinjiang Medical University, Urumqi, 830000, China; d College of Animal Science, South China Agricultural University, Guangzhou, 510642, China; e The Affiliated Guangdong Second Provincial General Hospital of Jinan University, Guangzhou, 510317, China

**Keywords:** Intergenerational family interactions, Older adults, Frailty, Cross-national comparison

## Abstract

**Objectives:**

This study aimed to examine the association between types of intergenerational family interactions and frailty index (FI) among older adults across countries with different cultural backgrounds, and to explore variations in these associations among older individuals.

**Methods:**

We conducted a comprehensive longitudinal, cross-cultural analysis, involving adults aged 60 years and older from four countries, each participating in a nationally representative longitudinal cohort study: the China Health and Retirement Longitudinal Study (CHARLS; Wave 1-Wave 4), the Korean Longitudinal Study of Aging (KLoSA; Wave 4-Wave 7), the Health and Retirement Study (HRS; Wave 11-Wave 14), and the English Longitudinal Study of Ageing (ELSA; Wave 7-Wave 9).

**Results:**

Associations varied across countries. In the U.S., greater time spent on grandchild care was significantly associated with a lower FI, whereas more frequent intergenerational contact was marginally associated with a lower FI only in China. Downward economic support was associated with a higher FI in China (amount) and in the U.S. and the U.K. (any transfer), while upward economic support was marginally associated with a higher FI in Korea (amount) and significantly associated with a higher FI in the U.S. (any transfer) but was not statistically significant in China.

**Discussion:**

These findings suggest that certain forms of intergenerational family interactions may be associated with lower frailty among older adults, warranting further investigation.

## Highlights

• Population ageing is accelerating, and frailty is an important public health concern among older adults.
• Grandchild caregiving is associated with a lower FI in the U.S., but not in China, Korea, or the U.K.
• More frequent intergenerational contact is marginally associated with a lower FI in China, while associations are not statistically significant in the other countries.
• Economic support—particularly downward support—is more often associated with a higher FI across several countries.

## 1 Introduction

With increased life expectancy, addressing frailty in older adults has become a pressing issue. The ideal goal of healthy aging is to extend life expectancy without worsening frailty. Frailty, an age-associated syndrome, is a key factor in the decline of physical, cognitive, and social functions and contributes to mortality among older adults (Li et al., 2018). Frailty is linked to higher risks of falls, hospitalizations, and delirium (Hoogendijk et al., 2019; Kojima et al., 2018) and has been shown to reduce quality of life (Vanleerberghe et al., 2019).

Various assessment tools, such as the Fried Frailty Phenotype (Fried et al., 2001), Tilburg Frailty Indicator (Gobbens et al., 2010), and the frailty index (FI), are widely used to measure frailty (De Witte et al., 2013; Kanters et al., 2017). Currently, frailty assessment tools typically encompass multiple dimensions, including older adults’ health status, cognitive function, mental well-being, sensory function (hearing and vision), activities of daily living, fatigue levels, disease resistance, chronic conditions, and social participation.

However, significant academic debate persists regarding the measurement dimensions, screening methods, evaluation criteria, and operational definitions of frailty, and a universally accepted assessment framework has yet to be established. Against this backdrop, frailty is increasingly understood as shaped not only by biomedical factors but also by social and relational resources, highlighting the need to examine family contexts as modifiable levers for healthy ageing.

Research suggests that frailty is modifiable; it can be improved, maintained, or worsened through interventions (Kojima, Liljas, & Iliffe, 2019). This insight has expanded research in frailty prevention and intervention, highlighting the value of understanding frailty triggers. Studies on frailty span diverse fields, including geriatric medicine, nursing, nutrition, and psychology (Gale et al., 2018; Kato et al., 2024; Kojima, Taniguchi, et al., 2019; Ni Lochlainn et al., 2021). However, research from a family sociological perspective remains limited.

In essence, family structure and intergenerational relationships may be crucial factors influencing frailty among older adults. This is particularly relevant in the context of population ageing, where limited social care and healthcare resources make family networks an essential supplementary support system (Yang et al., 2023). Consequently, intergenerational family interactions could be a potential determinant of frailty. Existing studies, such as those by Anantapong et al. (2020) and Liu et al. (2020), suggest that perceived family support may help reduce frailty rates. However, broader family-level factors remain underexplored.

Additionally, current research often uses cross-sectional data, and overlooks the initiative of older adults, viewing them primarily as care recipients. In fact, older parents globally often assist adult children, whether through financial support or grandchild care (Arpino & Bordone, 2014). Studies have explored how this altruism relates to older adults’ health (Wang et al., 2024) and cognitive function (Caputo et al., 2024), but the association with frailty remains underexplored. Therefore, further theoretical and empirical research is urgently needed to understand how intergenerational family interactions are associated with frailty in older adults.

However, existing evidence is often organized around a single support domain, making it difficult to assess their joint associations and to interpret why effects appear mixed across studies and settings. A growing body of work links intergenerational support and broader social relations to frailty and related health outcomes, but several limitations remain. First, different forms of support may operate through distinct—and sometimes opposing—mechanisms: grandchild care can provide role meaning and activity yet also create time and physical burdens (Arpino & Bordone, 2014; Caputo et al., 2024); perceived family support and social resources can be linked to lower frailty (Anantapong et al., 2020; Liu et al., 2020); financial transfers can signal solidarity but may also reflect need, dependence, or resource strain (Wang et al., 2024); and frequent contact can reduce loneliness and facilitate support but may vary in meaning across family structures and cultures (Yang et al., 2023). Second, many studies treat each support domain separately or rely on cross-sectional evidence, which makes it hard to distinguish independent from overlapping associations when multiple exchanges co-occur. Third, cross-national evidence remains limited, even though welfare and family regimes plausibly condition whether a given exchange is experienced as support, obligation, or strain.

Building on this literature, the present study advances the field in three ways. First, we simultaneously consider grandchild care, upward and downward economic transfers, and contact frequency in a single analytic framework, allowing us to assess their net associations with frailty when multiple exchanges coexist within families. Second, using harmonized longitudinal data from four nationally representative cohorts, we compare East Asian (China, Korea) and Western (United States, United Kingdom) contexts, thereby speaking directly to how welfare and family regimes may shape the health implications of intergenerational exchanges. Third, we examine heterogeneity by gender and by number of children, two dimensions that are central to caregiving expectations and resource allocation within families.

Substantively, our primary aim is to document how the pattern of associations between intergenerational exchanges and frailty varies across welfare and family regimes, rather than to search for a universally “protective” or “harmful” form of support. Within each country, we also assess whether each exchange is associated with lower versus higher frailty when modeled jointly. Accordingly, we pose the following research questions: (1) Are grandchild care, upward and downward transfers, and contact frequency associated with subsequent frailty among older adults? (2) Do the signs and significance of these associations differ across the four national contexts? and (3) Are associations heterogeneous by gender and number of children?

The following section develops hypotheses based on Role Enhancement Theory and Intergenerational Solidarity Theory to guide these analyses.

## 2 Theoretical framework and research hypotheses

Drawing on Role Enhancement Theory and Intergenerational Solidarity Theory, this study conceptualizes intergenerational family support as multidimensional exchanges that may be associated with frailty through both benefit pathways (resources, role meaning, emotional connection) and strain pathways (obligation, role overload, and resource depletion). Accordingly, the expected direction of association may differ across support types and across social and policy contexts.

### 2.1 Grandchild care and frailty in older adults

From the perspective of Role Enhancement Theory, engaging in multiple social roles provides individuals with greater social resources, strengthens their support networks, and enhances their sense of self-efficacy, ultimately benefiting both physical and mental health (Sieber, 1974). Applying this theory to intergenerational relationships, research suggests that older adults who participate in grandchild caregiving and other intergenerational activities reinforce their familial role identity and experience a sense of being needed, which in turn boosts their self-efficacy. This positive psychological experience not only helps buffer the negative effects of stress on health but also significantly reduces the risk of depression, thereby slowing frailty progression (Bi, 2022). However, when caregiving intensity surpasses a certain threshold, role conflict mechanisms may emerge and become dominant (Hughes et al., 2007). Specifically, excessive caregiving responsibilities or prolonged caregiving duration can lead to increased time pressure, poorer sleep quality, and a higher risk of chronic pain, all of which contribute to frailty progression (Burn & Szoeke, 2015; Li et al., 2021).

Hypothesis 1 Providing care for grandchildren is associated with lower frailty among older adults.

### 2.2 Intergenerational economic support and frailty in older adults

From the perspective of Intergenerational Solidarity Theory, emotional bonds, contact, and mutual support within families constitute key social resources that can shape older adults’ health (Bengtson & Roberts, 1991). Economic transfers are a central form of functional solidarity, yet their health consequences are not uniformly beneficial and may depend on transfer direction, voluntariness, and reciprocity. Upward economic support (children to parents) may relieve material hardship, reduce financial stress, and enhance economic security, thereby benefiting physical and mental well-being (Herrera et al., 2022; Huang & Fu, 2021; Zhou & Bai, 2022). At the same time, if such support is accompanied by reduced autonomy or perceived dependency, it may carry psychological costs that offset health gains (Ang & Malhotra, 2022).

Downward economic support (parents to children) can reinforce older adults’ sense of familial authority and intergenerational role identity, offering potential psychological benefits (Wang & Cheng, 2023). However, providing financial help may also deplete limited resources, heighten chronic financial strain, and crowd out health-promoting investments (e.g., healthcare utilization, nutrition, and physical activity), which could accelerate frailty progression. In settings where formal social protection is relatively limited, these resource-depletion dynamics may be more salient.

Taken together, intergenerational economic support may operate through both supportive and strain pathways, and existing evidence remains mixed regarding its net implications for frailty. Therefore, this study proposes the following hypothesis:

Hypothesis 2 Both the presence of intergenerational economic support (i.e., any downward or upward transfer) and the amount of such support are associated with greater frailty among older adults.

### 2.3 Frequency of intergenerational contact and frailty in older adults

Emotional closeness is another aspect of intergenerational relationships found to be a powerful predictor of support and care by virtue of the empathetic attachment it engenders (Hwang et al., 2022). According to Intergenerational Solidarity Theory, close and mutually respectful emotional bonds provide psychological support, help mitigate life stress, and contribute to the overall well-being of older adults (Hong et al., 2024). Empirical studies have shown that older adults who describe their relationship with their children as “very close” tend to exhibit significantly better mental health compared to those with more distant relationships (Yang et al., 2023). Conversely, strained intergenerational relationships can become a chronic stressor, increasing the risk of depression among older adults (Kim et al., 2020).

Importantly, such psychosocial benefits may also translate into physical resilience and lower frailty risk. Regular interaction with adult children constitutes a key indicator of “associational solidarity” and may reduce social isolation and loneliness—factors that are consistently linked to higher frailty risk in later life (Kojima et al., 2022; Politis et al., 2024). Moreover, more frequent contact can facilitate practical health management (e.g., monitoring functional changes, encouraging activity, and supporting timely care-seeking), thereby helping to slow frailty development (Mao et al., 2025; Zhang et al., 2025). Given the increasing prevalence of digital communication technologies—such as video calls, phone calls, text messages, emails, and social media interactions—which have redefined how intergenerational contact is maintained in contemporary society, and because our measure captures only these technology-mediated forms of interaction (excluding face-to-face contact), we conceptualize intergenerational contact in this study as the frequency of remote communication. Accordingly, this study proposes the following hypothesis:

Hypothesis 3 More frequent intergenerational contact is associated with lower frailty among older adults.

Life Course Theory emphasizes the impact of early life experiences and the long-term accumulation of advantages or disadvantages on individuals’ health outcomes (Elder et al., 2003). This theoretical framework provides an important foundation for understanding the longitudinal health effects of intergenerational relationships. The impact of intergenerational relationships on health exhibits significant heterogeneity, driven not only by key life course events (such as childhood adversity, marital status, etc.) but also by shifts in socio-cultural contexts (such as economic fluctuations and policy changes) (Buttery et al., 2015; Li et al., 2021). Therefore, this study incorporates key factors such as age, gender, living location, marital status, and family income as control variables in the analysis framework.

Particularly noteworthy is the one-child family structure in China, formed in response to the “population explosion crisis” in 1978. The intergenerational interaction patterns in these families differ significantly from those in multi-child families, yet existing research remains divided on this issue (Ding et al., 2019; Liu & Qi, 2020). Since the impact of the number of children on frailty has not been fully explored, this study will conduct a dedicated heterogeneity analysis on this aspect. Additionally, in terms of gender differences, research has found that social gender roles place women at a higher risk of falling into the “caregiver health dilemma” while men tend to benefit more from moderate intergenerational interactions (He & Wang, 2024; Hughes et al., 2007). This difference may be related to traditional cultural expectations about gender roles and gender-specific physiological stress responses. Therefore, this study will also conduct a heterogeneity analysis based on the gender of older adults.

In summary, the theoretical perspectives above yield three testable hypotheses concerning grandchild care, economic transfers, and contact frequency. Given that the cultural norms governing these exchanges differ markedly between East Asian societies, which emphasize familial cohesion and filial obligation, and Western societies, which prioritize individual autonomy, we expect the pattern of associations to vary across the four national contexts examined below.

## 3 Materials and methods

### 3.1 Sample selection

This analysis used data from the China Health and Retirement Longitudinal Study (CHARLS; Wave 1–Wave 4 (2011-2018), the Korean Longitudinal Study of Aging (KLoSA; Wave 4–Wave 7 (2012-2018), the Health and Retirement Study (HRS; Wave 11–Wave 14 (2012-2020), and the English Longitudinal Study of Ageing (ELSA; Wave 7–Wave 9 (2014-2020). Each study used a multi-stage probability sampling design and collected data on health, quality of life, economic status, family relationships, and intergenerational interactions. These studies are widely cited in gerontological research for their rigorous data collection and follow-up methods, providing reliable and comparable data (Brønnum-Hansen, 2014). The raw data were obtained via the Gateway to Global Aging Data platform. For further information on each survey, please refer to the Gateway to Global Aging Data website (http://gateway.usc.edu).

In this study, there were at least three survey waves in each country to ensure methodological consistency, comparability of data, and validity of findings. Participants in all cohorts were informed of their objectives, procedures, potential risks and benefits, and signed informed consent forms prior to participating in the surveys to ensure that they were aware of their rights, the voluntary nature of their participation, and the confidentiality of their data. These informed consent forms for each cohort were reviewed and approved by each regional ethics committee to ensure that they complied with international ethical standards. The initial sample sizes for CHARLS, KLoSA, HRS, and ELSA were 17,708, 5,289, 20,554, and 9,666, respectively. During the observation period, the numbers of participants who completed all survey waves were 11,988, 3,941, 12,440, and 6,900, corresponding to response rates of 67.70%, 74.51%, 60.52%, and 71.38%, respectively.

**Fig. 1:**
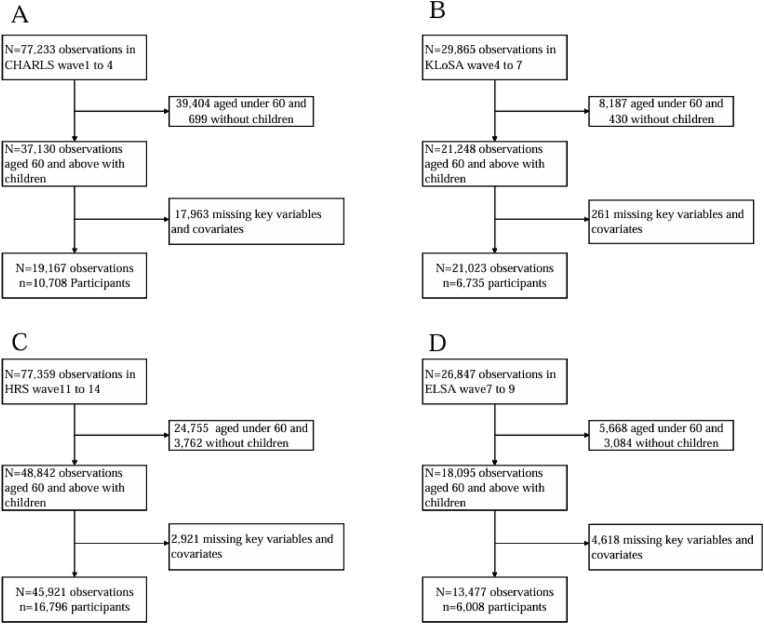
Study flowchart.

To maintain analytic consistency, we excluded participants under the age of 60, those without children, and performed listwise deletion for missing values on all variables of interest in the analysis. Health-related data such as cognitive function, daily activities, lifestyle, mental and physical health, and functional status were harmonized to allow FI calculation. To avoid sample size reduction due to panel attrition, all our analyses were conducted using unbalanced panel data. The final sample sizes were CHARLS (N = 19,167), KLoSA (N = 21,023), HRS (N = 45,921), and ELSA (N = 13,477). The sample selection process is shown in Fig. 1.

### 3.2 Variable measurement

#### 3.2.1 Frailty

Following previous studies (Kanters et al., 2017; Sun et al., 2024), this study assesses frailty using the FI. In CHARLS, HRS, and ELSA, the FI included diagnoses of hypertension, diabetes, heart disease, stroke, cancer, arthritis, lung disease, and mental illness; self-reported vision, hearing, and health; ADL/IADL limitations (e.g., dressing, bathing, eating, transferring from bed, toilet use, managing finances, taking medication, shopping, and meal preparation); mobility-related difficulties (e.g., walking 1 km, standing up after prolonged sitting, climbing stairs without resting, lifting heavy items, picking up a coin, stooping/kneeling/crouching, and raising arms above shoulder level); as well as depression and cognitive status. In KLoSA, memory-related disease and several mobility-related tasks (standing up after prolonged sitting, climbing stairs without resting, lifting heavy items, picking up a coin from a table, stooping/kneeling/crouching, and raising arms above shoulder level) were not available; therefore, we used difficulty going out for short distances to harmonize the walking item. Detailed definitions and coding rules for FI components are presented in Appendix Table A1.

#### 3.2.2 Intergenerational interaction

Intergenerational interaction includes the following variables: First, Intergenerational care hours, defined as the weekly hours older adults spend caring for grandchildren (log-transformed). Second, for the intergenerational economic support variable, researchers commonly use either the amount (von Saenger et al., 2025) or the presence/absence (Silverstein et al., 2025) as measures. Specifically, downward economic support, measured in CHARLS and KLoSA as the financial amount older adults provide to their children (log-transformed); in HRS and ELSA, where specific amounts are not recorded, it is defined as whether the older adults provide financial support to their children. For upward economic support, measured in CHARLS and KLoSA as the financial amount provided by children to older adults (log-transformed); in HRS, as whether children provide financial support to older adults, while ELSA lacks this variable. Finally, contact frequency, indicating how often older adults communicate with children via phone, text, email, or messaging apps, where a higher value represents higher contact frequency. Contact frequency is measured on a 1–10 scale for CHARLS and KLoSA (1 = no contact, 10 = almost daily) and a 1–7 scale for HRS and ELSA (1 = no contact, 7 = daily).

#### 3.2.3 Control variables

The study incorporates the following time-varying variables as control variables.: age, marital status (married = 1, other = 0), household residence location (rural = 1, urban = 0), health insurance (insured = 1, not insured = 0), pension (participating = 1, not participating = 0), number of children, co-residence with children (yes = 1, no = 0), and per capita household income (log-transformed).

Among these: (1) The ELSA lacks an urban-rural variable; (2) Health and pension insurance vary by survey: in HRS, health insurance refers to participation in any government or private health plan, and pension insurance includes receiving or participating in any pension plan; in KLoSA, health insurance indicates participation in the national health plan, and pension includes private, corporate, or government pension participation or receipt; in ELSA, universal health coverage is assumed, so health insurance is excluded, and pension is defined as participation in or receipt of any private or employer-sponsored pension; in CHARLS, health insurance includes participation in basic or commercial plans, and pension includes urban or rural pension plans. (3) HRS lacks co-residence data, so it is substituted with the proximity of children within 10 miles.

Differences in variable construction across the four cohorts are summarized in Appendix Table A2. Because the variable measures are constructed differently across cohorts, we do not directly compare the absolute magnitudes of coefficients across countries. Our focus is on the direction and statistical significance of the associations between “greater support/any transfer” and a higher or lower FI, rather than on precise cross-country rankings of effect sizes. To facilitate interpretation, we further translate key coefficients into realistic effect-size metrics: for log-transformed continuous variables, we report the predicted change in FI associated with a doubling of the original amount; for dichotomous indicators, we report the discrete 0–1 change; for contact frequency, we report the change associated with a one-point increase in the score (see Table 3).

### 3.3 Statistical analysis

First, we conducted descriptive statistics on the panel data from the four countries to illustrate sample characteristics. Second, the Hausman test rejected the random-effects specification in favor of fixed effects. We therefore employed a fixed-effects model with individual and wave fixed effects to analyze the association between intergenerational interaction variables and frailty, while adjusting for a range of time-varying individual- and family-level characteristics. Robust standard errors were clustered at the individual level.

Third, we performed five sensitivity analyses: (a) We replaced the dependent variable with a binary frailty status indicator (FI > 0.25), consistent with Li (2024) and Fan et al. (2020). (b) We applied 1% winsorization to the FI to mitigate the influence of extreme values. (c) Given that the Korean sample is constrained by data availability, the FI can only be constructed using 23 components. To ensure cross-country comparability and to test the robustness of our findings to the FI construction, we further reconstructed a simplified FI for the other countries using the same 23-component standard adopted for Korea and re-estimated the regression models accordingly. (d) To control for potential time-varying confounders and mitigate reverse causality concerns, we re-estimated the baseline models using lagged intergenerational support variables (FI_t ∼ transfers_{t-1}). (e) To evaluate potential bias from attrition and listwise deletion, we re-estimated the models using inverse probability weighting (IPW) (Seaman & White, 2013).

Specifically, we estimated the probability of being included in the baseline regression sample among respondents aged 60+ with children, using the pre-deletion sample; the model included all baseline control variables and wave indicators, and missing covariates were mean-imputed for the selection equation only. We constructed inverse probability weights as the inverse of the predicted inclusion probability and winsorized the weights at the 1st and 99th percentiles. We then re-estimated the fixed-effects models in the analytic sample using the trimmed probability weights.

Fourth, given the exploratory nature of cross-national comparisons and the potential loss of statistical power arising from the differences in variable construction discussed above, we retain the reporting of marginally significant associations (p < 0.10) but clearly distinguish them from statistically significant results using the † symbol throughout.

Finally, we conducted heterogeneity analysis by gender and number of children to examine group differences in the associations between intergenerational interaction and frailty. It should be noted that the multiple comparisons across four countries and subgroups may increase the risk of false positives. To address the issue of multiple testing, we applied the Bonferroni correction separately for each subgroup dimension (gender and number of children), as these represent two distinct, theoretically motivated analytical dimensions rather than a single joint analysis. Within each dimension, the correction divides by the number of tests per country (4 variables × 2 groups = 8; p = 0.05/8 = 0.00625).

All analyses were performed using Stata 17.0.

## 4 Results

### 4.1 General characteristics

As shown in Table 1, the average ages of the samples from China, Korea, the U.S., and the U.K. are 69, 72, 73, and 72 years, respectively. Except for China, where the proportion of males exceeds 50%, the ratios of males in Korea, the U.S., and the U.K. are lower than those of females. Notable differences exist in intergenerational interactions across countries. China has the highest average weekly hours of grandchild care (logarithmic; mean = 1.019), followed by the U.S. (mean = 0.363), with Korea (mean = 0.162) and the U.K. (mean = 0.091) having fewer hours. In terms of economic support, older adults in China both provide and receive more financial support than those in Korea. In the U.S. and U.K., the proportions of financial support given to children are 33.07% and 15.95%, respectively. Regarding contact frequency, China (mean = 8.482) is slightly lower than Korea (mean = 8.762), while the U.S. (mean = 5.043) has a lower frequency than the U.K. (mean = 6.219).

**Table 1:** Main baseline characteristics of participants.

Characteristics	China (N = 19167)	Korea (N = 21,023)	U.S. (N = 45,921)	U.K. (N = 13,477)
FI	0.219 ± 0.141	0.171 ± 0.123	0.253 ± 0.165	0.180 ± 0.132
Intergenerational care hours (logarithmic)	1.019 ± 1.658	0.162 ± 0.744	0.363 ± 0.913	0.091 ± 0.502
Amount of downward intergenerational economic support (logarithmic)	0.641 ± 2.132	0.065 ± 0.640		
Downward intergenerational economic support			15,187 (33.07)	2149 (15.95)
Amount of upward intergenerational economic support (logarithmic)	6.123 ± 3.505	1.312 ± 2.525		
Upward intergenerational economic support			3054 (6.65)	
Contact frequency	8.482 ± 2.123	8.762 ± 1.580	5.043 ± 2.714	6.219 ± 0.980
Age	68.595 ± 6.661	72.340 ± 8.173	72.814 ± 8.901	71.560 ± 7.543
Married, %	15,477 (80.75)	15187(72.24)	27,224 (59.28)	9545 (70.82)
Rural, %	13,984 (72.96)	5966(28.38)	12,913 (28.12)	
Pension, %	16,270 (84.89)	14,094 (67.04)	17,529 (38.17)	10,026 (74.39)
Health insurance, %	18,482 (96.43)	19,987 (95.07)	43,788 (95.36)	
Number of children	3.180 ± 1.463	3.222 ± 1.426	3.376 ± 1.962	2.517 ± 1.320
Co-residence with children, %	7268 (37.92)	6635 (31.56)	24,568 (53.50)	2113 (15.68)
Per capita household income (logarithmic)	9.173 ± 1.587	5.364 ± 2.677	10.442 ± 1.483	10.070 ± 0.879

### 4.2 Association between intergenerational family support and older adult frailty

Table 2 reports the baseline regression results. To facilitate interpretation of coefficient magnitudes, Table 3 further translates each intergenerational support variable into more interpretable realistic effect sizes. Detailed confidence intervals and the corresponding percentages of the sample mean FI are reported in Table 3. For log-transformed continuous variables, the effect size corresponds to a doubling of the original amount (β × ln(2)); for the binary support indicators in the U.S. and the U.K., the effect size corresponds to the discrete change from no support (0) to any support (1). For the contact frequency, the reported effect size corresponds to a one-point increase in the contact frequency score.

**Table 2:** Baseline regression results.

VARIABLES	FI			
	China N = 19,167	Korea N = 21,023	U.S. N = 45,921	U.K. N = 13,477
Intergenerational care hours	−0.000	−0.001	−0.001∗∗	0.001
	(0.001)	(0.001)	(0.001)	(0.001)
Amount of downward intergenerational economic support	0.001∗∗∗	0.001		
	(0.000)	(0.001)		
Downward intergenerational economic support			0.002∗∗	0.004∗∗
			(0.001)	(0.002)
Amount of upward intergenerational economic support	0.000	0.001†		
	(0.000)	(0.000)		
Upward intergenerational economic support			0.016∗∗∗	
			(0.002)	
Contact frequency	−0.001†	0.000	0.000	−0.000
	(0.001)	(0.001)	(0.000)	(0.001)
Constant	−0.084	−0.324∗∗∗	−0.374∗∗∗	0.475∗∗∗
	(1.162)	(0.029)	(0.086)	(0.175)
R-squared	0.167	0.075	0.146	0.081
Control Variables	YES	YES	YES	YES
Individuals FE	YES	YES	YES	YES
Year FE	YES	YES	YES	YES

*Note*: ① ∗∗∗p < 0.01, ∗∗p < 0.05, †p < 0.1. ② Robust standard errors in parentheses.

**Table 3:** Realistic effect sizes of intergenerational support on the FI.

	China	Korea	U.S.	U.K.
	N = 19,167	N = 21,023	N = 45,921	N = 13,477
Intergenerational care hours	−0.00004	−0.00061	−0.00074∗∗	0.00051
	(-0.00093, 0.00085)	(-0.00176, 0.00053)	(-0.00144, −0.00004)	(-0.00127, 0.00229)
Amount of downward intergenerational economic support	0.00080∗∗∗	0.00055		
	(0.00029, 0.00132)	(-0.00048, 0.00158)		
Downward intergenerational economic support			0.00228∗∗	0.00442∗∗
			(0.00017, 0.00440)	(0.00105, 0.00779)
Amount of upward intergenerational economic support	0.00019	0.00044†		
	(-0.00025, 0.00063)	(-0.00001, 0.00089)		
Upward intergenerational economic support			0.01573∗∗∗	
			(0.01101, 0.02037)	
Contact frequency	−0.00123†	0.00029	0.00005	−0.00049
	(-0.00252, 0.00005)	(-0.00093, 0.00150)	(-0.00031, 0.00041)	(-0.00274, 0.00176)

*Note*: ① ∗∗∗p < 0.01, ∗∗p < 0.05, †p < 0.1. ② 95% confidence intervals are reported in parentheses.

Regarding intergenerational care, only in the U.S., a doubling of weekly grandchild care hours is significantly associated with approximately a 0.00074 decrease in the FI, consistent with Hypothesis H1; the association is not statistically significant in China, Korea, or the U.K. For downward intergenerational economic support, in China, a doubling of the amount of downward transfers is significantly associated with a 0.00080 increase in the FI. Similarly, in the U.S. and the U.K., having downward intergenerational economic support is significantly associated with increases in the FI of 0.00228 and 0.00442 in the U.S. and the U.K. respectively, consistent with Hypothesis H2, whereas the association is not statistically significant in Korea.

For upward economic support, in the U.S., having upward intergenerational economic support is significantly associated with a 0.01573 increase in the FI. In Korea, a doubling of the amount of upward transfers is marginally significantly associated with a 0.00044 increase in the FI. The estimate in China is not statistically significant. Finally, in terms of intergenerational contact, in China, a one-point increase in the contact frequency score is marginally significantly associated with a 0.00123 decrease in the FI, providing suggestive evidence in the direction predicted by Hypothesis H3; in the other countries, the association between contact frequency and FI is not statistically significant.

### 4.3 Robustness test

To assess the robustness of the baseline results, we conducted five sensitivity tests. Three of these yield results broadly consistent with the baseline (Appendix Tables A3–A5): (a) replacing the continuous FI with a binary frailty indicator (FI > 0.25) and estimating a linear probability model; (b) winsorizing the FI at the 1st and 99th percentiles; and (c) reconstructing a simplified 23-item FI for cross-country comparability. In addition, we conducted two further analyses that more directly address identification concerns: lagged exposure models (Table 4) and inverse probability weighting (Table 5).

**Table 4:** Robustness test (d).

VARIABLES	FI			
	China N = 6290	Korea N = 15,504	U.S. N = 28,623	U.K. N = 7175
Intergenerational care hours	0.001	0.001	0.000	0.002
	(0.001)	(0.001)	(0.001)	(0.002)
Amount of downward intergenerational economic support	−0.001	0.000		
	(0.001)	(0.001)		
Downward intergenerational economic support	−0.000	−0.000		
	(0.001)	(0.000)		
Amount of upward intergenerational economic support			−0.001	−0.001
			(0.001)	(0.003)
Upward intergenerational economic support			0.001	
			(0.003)	
Contact frequency	−0.001	0.001	−0.001∗∗∗	0.002
	(0.001)	(0.001)	(0.000)	(0.002)
Constant	1.535	−0.502∗∗∗	−0.272∗∗	0.010
	(4.164)	(0.036)	(0.116)	(0.298)
R-squared	0.145	0.074	0.107	0.055
Control Variables	YES	YES	YES	YES
Individuals FE	YES	YES	YES	YES
Year FE	YES	YES	YES	YES

*Note*: ① ∗∗∗p < 0.01, ∗∗p < 0.05, †p < 0.1. ② Robust standard errors clustered at the individual level are reported in parentheses.

**Table 5:** Robustness test (e).

VARIABLES	FI			
	China N = 19,167	Korea N = 21,023	U.S. N = 45,921	U.K. N = 13,477
Intergenerational care hours	0.000	−0.001	−0.001∗∗	0.001
	(0.001)	(0.001)	(0.001)	(0.001)
Amount of downward intergenerational economic support	0.001∗∗	0.001		
	(0.001)	(0.001)		
Downward intergenerational economic support			0.002∗∗	0.005∗∗
			(0.001)	(0.002)
Amount of upward intergenerational economic support	0.000	0.001†		
	(0.001)	(0.000)		
Upward intergenerational economic support			0.016∗∗∗	
			(0.002)	
Contact frequency	−0.001	0.000	0.000	−0.001
	(0.001)	(0.001)	(0.000)	(0.001)
Constant	−0.001	−0.324∗∗∗	−0.380∗∗∗	0.462∗∗
	(0.001)	(0.029)	(0.088)	(0.187)
R-squared	0.861	0.803	0.901	0.925
Control Variables	YES	YES	YES	YES
Individuals FE	YES	YES	YES	YES
Year FE	YES	YES	YES	YES

*Note*: ① ∗∗∗p < 0.01, ∗∗p < 0.05, †p < 0.1. ② Robust standard errors clustered at the individual level are reported in parentheses.

Table 4 presents the results of the lagged exposure analysis. Compared with the baseline simultaneous models (FI_t ∼ transfers_t), the lagged models (FI_t ∼ transfers_{t-1}) yield largely attenuated and non-significant associations, with the exception of contact frequency in the U.S. (p < 0.01). The attenuation in significance may be attributable to the high variability of intergenerational interactions between parents and children across waves, combined with the relatively long intervals between survey waves (e.g., two years in CHARLS), which make it difficult for the lagged models to capture the effects of intergenerational support on frailty. However, this does not suggest that the baseline associations are driven by reverse causality. Rather, it indicates that the relationship between intergenerational support and frailty operates primarily through contemporaneous pathways.

Table 5 presents the results using inverse probability weighting. The results are broadly consistent with the baseline findings. Downward intergenerational economic support remains positively associated with the FI in China (p < 0.05), the U.S. (p < 0.05), and the U.K. (p < 0.05), and upward intergenerational economic support remains positively associated with the FI in the U.S. (p < 0.01). Intergenerational care hours remain negatively associated with the FI in the U.S. (p < 0.05).

### 4.4 Heterogeneity analysis

The heterogeneity analysis results are shown in Appendix Tables A6–A9, with associations surviving the Bonferroni correction (p < 0.00625) shown in bold.

By gender, intergenerational interactions tend to be more strongly associated with the FI in older females. Specifically, in China and the U.S., higher downward intergenerational economic support is associated with a higher FI among older females (β = 0.002, p < 0.01; β = 0.004, p < 0.01), whereas the corresponding associations are not statistically significant among older males. In Korea, higher downward economic support is marginally significantly associated with a higher FI among older females (β = 0.003, p < 0.1), and higher upward economic support is significantly associated with a higher FI among older females (β = 0.001, p < 0.01), while neither association is statistically significant among older males. By contrast, in the U.K., the presence of downward economic support is associated with a higher FI among older males (β = 0.006, p < 0.01) but is not statistically significant among older females (β = 0.003, p > 0.1). In the U.S., the presence of upward intergenerational economic support is associated with a higher FI for both males and females (β = 0.016, p < 0.01).

Regarding the number of children, in China and Korea, the association between downward economic support and frailty is more pronounced among older adults with only one child. In China, although downward economic support is associated with a higher FI in both groups, the coefficient is larger in the one-child group (β = 0.002) than in the multiple-children group (β = 0.001). In Korea, downward economic support is marginally significantly associated with a lower FI among older adults with only one child (β = −0.006, p < 0.1), while the association is not statistically significant among those with multiple children. In contrast, the U.S. and the U.K. exhibit a clearer pattern in which the presence of downward economic support is associated with a higher FI among older adults with multiple children (β = 0.003, p < 0.05; β = 0.004, p < 0.05), whereas the corresponding estimates for the one-child group are not statistically significant. Additionally, in the U.S., higher intergenerational care hours are associated with a lower FI among older adults with multiple children (β = −0.001, p < 0.05). The presence of upward economic support is associated with a higher FI in both child-number groups in the U.S., with a larger magnitude among those with only one child (β = 0.033, p < 0.01).

After correction, the associations between downward economic support and FI among older females in China and the U.S., and between upward economic support and FI among older females in Korea, remain significant. In the U.S., the association between upward economic support and a higher FI survives the correction across all gender and child-number subgroups. Other associations that were significant at conventional levels do not survive the correction and should be interpreted with caution. Nonetheless, the core patterns identified in the heterogeneity analyses remain supported after accounting for multiple testing.

## 5 Discussion

As family sizes decrease and life expectancy continues to rise, understanding how different forms of intergenerational interaction are associated with frailty in older adults becomes increasingly important. In this context, we aim to contribute new evidence to discussions on different forms of intergenerational interaction and their associations with frailty in older adults. Descriptive statistics from the four countries reveal significant differences in intergenerational interaction behaviors. Although China and Korea share a Confucian cultural background, older adults in China spend more time caring for grandchildren and provide and receive more financial support compared to their counterparts in Korea. This aligns with findings by Zhang and Liu (2022), which highlight stronger family values in China, where intergenerational interactions are essential for family continuity and inheritance.

In contrast, in Korea, intergenerational interactions are often viewed as optional (Ko & Hank, 2014). For the Western countries, the U.S. shows higher levels of grandchild caregiving and financial support to children than the U.K. However, despite its lower prevalence, grandchild caregiving is still present in the U.K. Wellard (2011) found that grandparental caregiving often complements market or public childcare services. Among U.K. grandparents with grandchildren under 16 years, 17% provide at least 10 h of intensive childcare per week, and approximately 1 in 30 serves as a full-time caregiver. This indicates that, over the past decade, grandparents globally have increasingly contributed to raising and educating grandchildren (Buchanan & Rotkirch, 2018).

Interestingly, patterns in remote intergenerational contact move in the opposite direction from grandchild care and financial exchanges: contact is slightly less frequent in China than in Korea, and in the U.S. than in the U.K. One plausible explanation is substitution via in-person access. In our descriptive statistics, 37.9% of older adults in China live with an adult child, and nearly half of U.S. older adults live close to their children. Because the contact measure refers to non co-residing children, higher co-residence or geographic proximity often linked to grandchild caregiving and financial exchanges may reduce the need to maintain frequent remote contact with other children, especially in larger families (Pezzin et al., 2015). Co-residence itself is also dynamic over the life course and varies by gender and urban-rural context, underscoring that “living together” is not a fixed arrangement (Chang, 2025).

Within multi-child families, several mechanisms can further dampen contact with non-coresident children. Siblings may free-ride when one child co-resides or lives nearby, leading others to scale back contact (Jahangir et al., 2025; Maruyama & Johar, 2017). In addition, co-residing older adults—particularly those providing intensive grandchild care—may have less time and energy to sustain frequent communication with other children (Arpino & Bordone, 2017). Finally, digital communication can partly compensate when face-to-face contact is constrained, although uptake and meaning of digital “solidarity” vary across cultural and policy contexts (Fu et al., 2025).

Building on these descriptive patterns, this study identifies several key findings regarding the associations between intergenerational interactions and frailty in older adults, which we discuss in relation to our guiding hypotheses.

Regarding our first hypothesis on grandchild caregiving, we find that intergenerational care hours are significantly associated with a lower FI in the U.S., whereas the corresponding estimates are not statistically significant in China, Korea, or the U.K. We hypothesize that caregiving activities, which require physical involvement (such as accompanying and caring for grandchildren), may contribute to this association. Specifically, caregiving could be linked to higher levels of physical activity, potentially supporting the maintenance of muscle strength and balance, and thus be associated with slower frailty progression. Although this mechanism remains to be tested, it is consistent with prior research suggesting that caregiving provides opportunities for social interaction, which has been associated with a lower risk of cognitive decline (Jun, 2015; Arpino & Bordone, 2014). Grandchild caregiving may also strengthen the relationship between older adults and their children, which could be linked to higher self-efficacy and better mental health (Shen & Zhang, 2020), and in turn, to lower levels of frailty. In sum, the protective association of grandchild care with frailty observed in the U.S.---but not in the other three countries---suggests that the health implications of this form of support may be context-dependent, potentially shaped by differences in welfare regimes, gender norms, and the availability of formal childcare.

Turning to our second hypothesis concerning intergenerational economic support, we find that the associations differ by transfer direction and country, offering partial support for the hypothesis that economic support is associated with greater frailty. Downward economic support is associated with a higher FI in China (amount), and it is also positively associated with FI in the U.S. and the U.K. (any transfer). For upward economic support, a marginally significant positive association with FI is observed in Korea (amount) and in the U.S. (any transfer), whereas the estimate in China is not statistically significant. One hypothesized mechanism, which our data do not directly test, is that receiving support could foster greater dependence and feelings of helplessness, as well as reduced social engagement, which may be linked to higher frailty. This is consistent with Peng et al. (2019), who found that older adults tend to report lower levels of self-esteem and independence when receiving financial support, which may be related to lower overall well-being. Moreover, providing financial support may deplete resources that older adults would otherwise use to maintain their own health, potentially being associated with higher frailty. Collectively, these findings suggest that both providing and receiving financial support can be associated with higher frailty, though the specific direction of transfer that emerges as salient varies across national contexts, likely reflecting differences in social security systems and cultural expectations regarding filial obligations.

With respect to our third hypothesis on intergenerational contact, the data from China indicate that higher contact frequency with children is marginally associated with a lower FI, providing partial support for the hypothesis that more frequent contact is associated with lower frailty. This finding suggests that maintaining emotional connections with children may be linked to better health outcomes for older adults in certain contexts. This marginally significant association found only in the Chinese context may reflect the particular importance of intergenerational contact within Confucian cultural frameworks (Gu et al., 2023). According to the dual filial piety model, frequent contact serves both as an expression of filial piety itself and as a mechanism through which both generations reinforce the parent-child bond (Bedford & Yeh, 2019). In contrast, older adults in Western societies tend to maintain greater independence and can draw upon broader social networks, such as friends, community organizations, and formal support services, to meet their emotional needs (Schwarzbach et al., 2014). This diversified support structure may reduce reliance on contact with adult children, thereby attenuating the protective association of such contact with lower frailty. Additionally, cross-national differences in healthcare systems may moderate the relationship between intergenerational contact and frailty (Chen et al., 2024). Nevertheless, as older adults’ social networks contract with age, they may increasingly seek interaction with their children to alleviate feelings of loneliness (Li & Guo, 2022). Thus, maintaining positive intergenerational contact remains relevant for older adults' well-being. In summary, the protective association of contact frequency appears most salient in the Chinese context, highlighting how cultural frameworks may shape the health relevance of emotional bonds with children.

The robustness of our findings was supported by multiple sensitivity analyses. The key associations remained consistent when using a binary frailty indicator, after winsorizing the FI, and when applying a simplified 23-item FI for cross-country comparability. Inverse probability weighting analyses confirmed that attrition and missing data did not substantially bias the results. However, the lagged exposure models yielded attenuated associations, suggesting that the relationship between intergenerational support and frailty operates primarily through contemporaneous pathways rather than long-lagged effects, and that reverse causality cannot be entirely ruled out.

Having established the main patterns of association, we examined whether these relationships vary by gender and number of children, as posited in our heterogeneity analyses. The heterogeneity analysis by gender reveals that the associations between economic support and FI vary substantially across countries, reflecting differences in socioeconomic environments, gender norms, labor force participation patterns, and pension systems. Specifically, economic support tends to be more strongly associated with the FI among older females in China, Korea, and the U.S. Due to differences in physiological conditions, women generally have longer life expectancies. Additionally, gender-based division of labour often results in women taking on more household responsibilities. As a result, they may tend to rely more on intergenerational support and place greater focus on their children than men, which may amplifiy the associations between intergenerational interactions and frailty within this group. These findings suggest that women may be more susceptible to both the benefits and strains embedded in economic exchanges with their children.

In contrast, the U.K. shows an opposite pattern for downward economic support. Specifically, such support is associated with higher FI among older males, but not among females. Nevertheless, this pattern is broadly consistent with recent evidence from a longitudinal study based on U.K. survey data (Cui et al., 2025). This study demonstrates that traditional gender division of labor, particularly increased income contribution, serves a protective function for British men's psychological health; conversely, diminished financial contribution may undermine their wellbeing. This gendered pattern in the U.K. underscores the need to consider how macroeconomic and policy contexts interact with traditional gender roles to shape the health implications of intergenerational support. Taken together, findings across these countries suggest that frailty prevention for women warrants particular attention (Bernabei et al., 2022).

Regarding the heterogeneity in the number of children, in China and Korea—both East Asian societies deeply influenced by Confucian norms of filial piety—the association between downward economic support and frailty is more pronounced among older adults with only one child. Mutual assistance among multiple children can alleviate caregiving burdens and reduce economic dependence on parents. Additionally, having more children helps prevent older parents from suppressing their own needs due to concerns about overburdening an only child with excessive financial demands. This pattern suggests that in Confucian contexts, having multiple children may buffer the resource-depletion effects of providing financial support.

Interestingly, although both China and Korea belong to the same East Asian Confucian cultural sphere, they exhibit a clear difference in how downward intergenerational support is associated with frailty among older adults in one-child families, which may stem from variations in social security systems and reciprocal expectations. In Korea, continuous or uninterrupted grandparenting by older parents for their adult children is an inherent feature of Korean family values and intergenerational relationships; however, older parents are often psychologically prepared to accept a 20-30 year time lag in expecting reciprocal support from their children, while also placing hope in improvements to the government welfare system to alleviate poverty in old age (Park & Lim, 2025). In contrast, older parents in China—particularly those in rural areas—receive relatively lower pension benefits and are more inclined to provide financial support to their only child out of altruistic motives without expecting returns. Such unidirectional economic transfers may further deplete their already limited resources, thereby contributing to greater frailty (Qiu et al., 2022). Thus, even within a shared cultural tradition, differences in social policy and welfare infrastructure can lead to divergent health outcomes associated with family support.

In the U.K. and the U.S., influenced by individualistic cultural norms, the relationship between older adults and their children is largely voluntary and reciprocal. Providing financial support to multiple children can place a greater strain on older parents’ resources, potentially exacerbating frailty. These findings in Western contexts highlight that when family support is more voluntary and reciprocal, the number of children may interact with support exchanges in ways that differ markedly from East Asian settings.

This study makes several contributions. First, it is the first to examine the association between various forms of family intergenerational support and the comprehensive health indicator of frailty in older adults from a social science perspective, opening new avenues for exploring potential intervention strategies that may be relevant to frailty. Second, this study highlights the need for government attention to intergenerational family interactions, suggesting that policies fostering intergenerational cohesion may warrant consideration, given the observed associations with frailty. Third, this study's findings can facilitate cross-disciplinary collaboration, encouraging deeper research in sociology, psychology, medicine, and related fields. Interdisciplinary studies can may contribute to a more comprehensive understanding of the nature and factors influencing frailty in older adults, enabling more effective solutions (Archibald et al., 2017). Finally, we used cohort data from four countries, each with a rigorous design and large sample size. All models controlled for individual and family-level variables, and sensitivity analyses were conducted on the baseline regression, strengthening the reliability of our conclusions.

Despite this contribution, our study has certain limitations. First, although the fixed-effects specification controls for time-invariant confounders, it cannot fully address time-varying confounders such as health shocks, income changes, or changes in children's employment status. Moreover, while we re-estimated the baseline models using lagged exposure variables to mitigate reverse causality, this approach cannot fully resolve this concern. Our findings should therefore be interpreted as describing longitudinal associations rather than identifying causal effects. Second, there are slight differences in questionnaire design and variable measurement methods across the cohort data from the four countries, which may affect the robustness of cross-national analysis results. As a result, we can only compare the significance of the associations between intergenerational support and frailty across countries, without directly comparing the effect sizes of intergenerational support between cohorts. Third, given the relatively recent baseline data and shorter follow-up periods for some countries (e.g., CHARLS began in 2011 with only four waves of data), we aligned the observation periods by analyzing similar years across the other three countries. This approach may affect the validity of estimates in other cohorts. Future studies should conduct long-term tracking of older adults to systematically observe changes in frailty over time. Fourth, we focused only on the independent associations of different types of intergenerational support on frailty, without analyzing factors such as satisfaction with intergenerational relationships or the emotional quality of these relationships (e.g., closeness, conflict levels). Future research should explore how various dimensions of intergenerational relationships relate to frailty in older adults. Fifth, our study did not examine the underlying mechanisms between intergenerational support and frailty (e.g., social isolation, subjective well-being, self-efficacy). Future studies should incorporate these factors to thoroughly investigate the mechanisms shaping the association between intergenerational relationships on frailty. Sixth, the heterogeneity analyses by gender and number of children were exploratory in nature, conducted to probe potential differential effects of intergenerational interactions on frailty across subgroups. Given the multiple testing involved, the evidence for a small portion of these results is insufficient to support strong conclusions after the Bonferroni correction. These patterns should therefore be treated as suggestive rather than definitive, and further exploration in subsequent analyses is needed to verify their consistency.

It should be noted that the mechanisms discussed above remain hypothetical. Our study identifies associations but does not directly test these pathways. Future research using mediation analysis or experimental designs is needed to establish the causal mechanisms underlying these associations.

## 6 Conclusions

This study compared differences in family intergenerational interactions across various cultural backgrounds. After harmonizing measures of intergenerational support in adults aged 60+ across four nations represented in longitudinal studies, we further explored the relationship between these interactions and frailty, focusing on the direction of association and variation in findings by country. Overall, the association between intergenerational interactions and the FI is not consistent across countries. In some countries, intergenerational support is associated with a lower FI (e.g., greater grandchild caregiving in the U.S. and, to a lesser extent, more frequent intergenerational contact in China), whereas financial transfers—especially downward transfers and, in certain contexts, upward transfers—are more often associated with a higher FI. These patterns highlight the importance of interpreting intergenerational support in light of each country's cultural context, family system, and policy environment.

## CRediT authorship contribution statement

**Wenze Tian:** Formal analysis, Data curation, Conceptualization. **Xiaoyu Wang:** Formal analysis, Data curation, Conceptualization. **Gang Hu:** Writing – review & editing, Funding acquisition. **Zheng-Hui Zhao:** Writing – review & editing, Supervision, Software, Project administration, Methodology, Funding acquisition.

## Ethical statement

Not applicable. (For this study, ethical approval was not deemed necessary as the CHARLS, KLoSA, HRS and ELSA data are publicly accessible. Access to these data requires a registration application via the Gateway to Global Aging Data website (http://gateway.usc.edu).

## Clinical trial number

Not applicable.

## Funding

The National Natural Science Foundation of China (Project No. 82401895). The Ministry of Education Humanities and Social Sciences Fund, Young Scholars Fund Project, 23XJJC840001.

## Declaration of competing interest

All authors disclosed no relevant relationships.

## Data Availability

The authors do not have permission to share data.
