# Supplementary material for: Intergenerational family support and older adult frailty: findings from four prospective cohort studies
Source: SSM Popul Health. 2026 May 30;34:101934. doi: 10.1016/j.ssmph.2026.101934 (PMC13266220; doi:10.1016/j.ssmph.2026.101934)
Supplement: Multimedia component 1 [file mmc1.docx]

Appendix

Table A1 Detailed definitions and coding rules for FI components

| No. | FI component | Description | | | | Coding |
| --- | --- | --- | --- | --- | --- | --- |
|  |  | **CHARLS** | **KLoSA** | **HRS** | **ELSA** |  |
| 1 | Hypertension | Have been diagnosed with hypertension | | | | Yes = 1, No = 0 |
| 2 | Diabetes | Have been diagnosed with diabetes | | | | Yes = 1, No = 0 |
| 3 | Heart disease | Have been diagnosed with heart disease | | | | Yes = 1, No = 0 |
| 4 | Stroke | Have been diagnosed with stroke | | | | Yes = 1, No = 0 |
| 5 | Cancer | Have been diagnosed with cancer | | | | Yes = 1, No = 0 |
| 6 | Arthritis | Have been diagnosed with arthritis | | | | Yes = 1, No = 0 |
| 7 | Lung disease | Have been diagnosed with lung disease | | | | Yes = 1, No = 0 |
| 8 | Mental illness | Have been diagnosed with mental illness | | | | Yes = 1, No = 0 |
| 9 | Memory-related disease | Have been diagnosed with memory-related disease |  | Have been diagnosed with memory-related disease | Have been diagnosed with memory-related disease | Yes = 1, No = 0 |
| 10 | Vision | Self- reported eyesight | | | | Excellent = 0, good = 0.25, fair = 0.5, poor = 0.75, very poor = 1 |
| 11 | Hearing | Self- reported hearing | | | | Excellent = 0, good = 0.25, fair = 0.5, poor = 0.75, very poor = 1 |
| 12 | Health | Self- reported health | | | | Excellent = 0, good = 0.25, fair = 0.5, poor = 0.75, very poor = 1 |
| 13 | Dressing | Have any difficulty with Dressing | | | | Yes = 1, No = 0 |
| 14 | Bathing | Have any difficulty with Bathing | | | | Yes = 1, No = 0 |
| 15 | Eating | Have any difficulty with Eating | | | | Yes = 1, No = 0 |
| 16 | Transferring from bed | Have any difficulty with Transferring from bed | | | | Yes = 1, No = 0 |
| 17 | Toilet use | Have any difficulty with using the toilet | | | | Yes = 1, No = 0 |
| 18 | Managing finances | Have any difficulty with Managing finances | | | | Yes = 1, No = 0 |
| 19 | Taking medication | Have any difficulty with Taking medication | | | | Yes = 1, No = 0 |
| 20 | Shopping | Have any difficulty with shopping | | | | Yes = 1, No = 0 |
| 21 | Meal preparation | Have any difficulty with preparing hot meals | | | | Yes = 1, No = 0 |
| 22 | Walking | Have any difficulty with Walking 1 km | Have any difficulty with going out for short distances | Have any difficulty with Walking 1 km | Have any difficulty with Walking 1 km | Yes = 1, No = 0 |
| 23 | Standing up after prolonged sitting | Have any difficulty with getting up from a chair after sitting for a long period |  | Have any difficulty with getting up from a chair after sitting for a long period | Have any difficulty with getting up from a chair after sitting for a long period | Yes = 1, No = 0 |
| 24 | Climbing stairs without resting | Have any difficulty with climbing several flights of stairs without resting |  | Have any difficulty with climbing several flights of stairs without resting | Have any difficulty with climbing several flights of stairs without resting | Yes = 1, No = 0 |
| 25 | Lifting heavy items | Have any difficulty with Lifting heavy items |  | Have any difficulty with Lifting heavy items | Have any difficulty with Lifting heavy items | Yes = 1, No = 0 |
| 26 | Picking up a coin from a table | Have any difficulty with Picking up a coin from a table |  | Have any difficulty with Picking up a coin from a table | Have any difficulty with Picking up a 5p coin from a table | Yes = 1, No = 0 |
| 27 | Stooping/kneeling/ crouching | Have any difficulty with stooping, kneeling, or crouching |  | Have any difficulty with stooping, kneeling, or crouching | Have any difficulty with stooping, kneeling, or crouching | Yes = 1, No = 0 |
| 28 | Raising arms above shoulder level | Have any difficulty with Raising arms above shoulder level |  | Have any difficulty with Raising arms above shoulder level | Have any difficulty with Raising arms above shoulder level | Yes = 1, No = 0 |
| 29 | Depression | 10-item CESD scale score, ranging from 0-30 | | 8-item CESD scale score, ranging from 0-10 | | For CHARLS and KLoSA, scores less than 10 = 0, otherwise = 1; For HRS and ELSA, scores less than 2 = 0, otherwise = 1 |
| 30 | Cognitive status | Word recall score + Date recognition score + Serial 7's test score | | | | Continuous variable, ranging from 0 to 1 |

Table A2. Differences in Variable Construction Across Four Cohorts

| **Variable** | CHARLS (China) | KLoSA (Korea) | HRS (U.S.) | ELSA (U.K.) |
| --- | --- | --- | --- | --- |
| Downward economic support | Amount, Continuous (log) | Amount, Continuous (log) | Whether, Binary (0/1) | Whether,  Binary (0/1) |
| Upward economic support | Amount, Continuous (log) | Amount, Continuous (log) | Whether, Binary (0/1) | Not available |
| Contact frequency | 1–10 scale (1 = no contact, 10 = almost daily) | 1–10 scale (1 = no contact, 10 = almost daily) | 1–7 scale (1 = no contact, 7 = daily) | 1–7 scale (1 = no contact, 7 = daily) |
| Co-residence | Direct measure, binary (0/1) | Direct measure, binary (0/1) | Proximity proxy (children within 10 miles) | Direct measure, binary (0/1) |
| Urban-rural | Binary (0/1) | Binary (0/1) | Binary (0/1) | Not available |
| Health insurance | Basic or commercial plans | National health plan | Any government or private plan | Not included (universal coverage) |
| Pension insurance | Urban or rural pension plans | Private, corporate, or government pension | Any pension plan | Private or employer-sponsored pension |

In Table A3, we replace the continuous FI with a binary frailty indicator (FI > 0.25) and estimate a linear probability model. For log-transformed continuous variables, coefficients are transformed using β × ln(2) to represent the percentage-point change in the probability of being frail associated with a doubling of the original amount; for binary and ordinal variables, coefficients are directly interpretable as percentage-point changes Unlike the baseline analysis, we report these transformed estimates directly in the text rather than in a separate table. Intergenerational care hours are not significantly associated with the probability of frailty in any of the four countries. For downward intergenerational economic support, a doubling amount of such transfers is marginally significantly associated with a higher probability of frailty in China (0.2 percentage points) and Korea (0.4 percentage points). In the U.K., providing downward intergenerational economic support is also marginally significantly associated with a higher probability of frailty (1.4 percentage points), whereas this association is not significant in the U.S. For upward intergenerational economic support, providing such support is significantly associated with a higher probability of frailty in the U.S. (3.3 percentage points), but this association is not significant in the other countries. Regarding contact frequency, a one-point increase in the contact frequency score is marginally significantly associated with a higher probability of frailty in the U.S. (0.2 percentage points), but this association is not significant in the other countries.

Given that the binary frailty indicator only captures whether an individual crosses the 0.25 threshold, intergenerational support variables, which tend to fluctuate across waves and exert relatively modest incremental effects on frailty, are more readily detected through the continuous FI. This likely explains why some associations that were statistically significant in the baseline model are only marginally significant in the binary specification. Nevertheless, the direction of the key coefficients remains broadly consistent across both models, supporting the robustness of the baseline findings.

Table A3. Robustness Test (a)

|  | Frailty status | | | |
| --- | --- | --- | --- | --- |
| VARIABLES | China  N=19,167 | Korea  N=21,023 | U.S.  N=45,921 | U.K.  N=13,477 |
| Intergenerational care hours | -0.002 | -0.001 | -0.003 | 0.001 |
|  | (0.003) | (0.003) | (0.003) | (0.007) |
| Amount of downward intergenerational economic support | 0.003† | 0.006† |  |  |
|  | (0.002) | (0.003) |  |  |
| Downward intergenerational economic support |  |  | 0.007 | 0.014† |
|  |  |  | (0.005) | (0.008) |
| Amount of upward intergenerational economic support | -0.000 | 0.001 |  |  |
|  | (0.001) | (0.001) |  |  |
| Upward intergenerational economic support |  |  | 0.033*** |  |
|  |  |  | (0.009) |  |
| Contact frequency | -0.003 | 0.002 | 0.002† | 0.001 |
|  | (0.003) | (0.002) | (0.001) | (0.005) |
| Constant | -0.763 | -1.020*** | -0.725** | 1.880** |
|  | (3.609) | (0.103) | (0.346) | (0.777) |
| R-squared | 0.081 | 0.033 | 0.045 | 0.023 |
| Control Variables | YES | YES | YES | YES |
| Individuals FE | YES | YES | YES | YES |
| Year FE | YES | YES | YES | YES |

*Note*: ① *** p<0.01, ** p<0.05, † p<0.1. ② Robust standard errors clustered at the individual level are reported in parentheses.

Table A4 presents the results of the winsorised FI at the 1st and 99th percentiles. The coefficients and significance levels of the key variables are virtually identical to the baseline estimates across all four countries, confirming the robustness of the baseline findings.

Table A4. Robustness Test (b)

|  | FI (1% winsorised) | | | |
| --- | --- | --- | --- | --- |
| VARIABLES | China  N=19,167 | Korea  N=21,023 | U.S.  N=45,921 | U.K.  N=13,477 |
| Intergenerational care hours | -0.000 | -0.001 | -0.001** | 0.001 |
|  | (0.001) | (0.001) | (0.001) | (0.001) |
| Amount of downward intergenerational economic support | 0.001*** | 0.001 |  |  |
|  | (0.000) | (0.001) |  |  |
| Downward intergenerational economic support |  |  | 0.002** | 0.004** |
|  |  |  | (0.001) | (0.002) |
| Amount of upward intergenerational economic support | 0.000 | 0.001† |  |  |
|  | (0.000) | (0.000) |  |  |
| Upward intergenerational economic support |  |  | 0.016*** |  |
|  |  |  | (0.002) |  |
| Contact frequency | -0.001† | 0.000 | 0.000 | -0.000 |
|  | (0.001) | (0.001) | (0.000) | (0.001) |
| Constant | -0.051 | -0.314*** | -0.357*** | 0.424** |
|  | (1.144) | (0.028) | (0.085) | (0.170) |
| R-squared | 0.167 | 0.076 | 0.146 | 0.080 |
| Control Variables | YES | YES | YES | YES |
| Individuals FE | YES | YES | YES | YES |
| Year FE | YES | YES | YES | YES |

*Note*: ① *** p<0.01, ** p<0.05, † p<0.1. ② Robust standard errors clustered at the individual level are reported in parentheses.

Table A5 presents the results using the simplified 23-item FI. The key associations remain consistent: the amount of downward intergenerational economic support is associated with a higher FI in China (p<0.05), downward intergenerational economic support is associated with a higher FI in the U.K. (p<0.05), upward intergenerational economic support is associated with a higher FI in the U.S. (p<0.01), and intergenerational care hours are associated with a lower FI in the U.S. (p<0.05).

Table A5. Robustness Test (c)

|  | 23-item FI | | |
| --- | --- | --- | --- |
| VARIABLES | China  N=19,167 | U.S.  N=45,921 | U.K.  N=13,477 |
| Intergenerational care hours | -0.000 | -0.001** | 0.001 |
|  | (0.001) | (0.000) | (0.001) |
| Amount of downward intergenerational economic support | 0.001** |  |  |
|  | (0.000) |  |  |
| Downward intergenerational economic support |  | 0.001 | 0.004** |
|  |  | (0.001) | (0.001) |
| Amount of upward intergenerational economic support | 0.000 |  |  |
|  | (0.000) |  |  |
| Upward intergenerational economic support |  | 0.013*** |  |
|  |  | (0.002) |  |
| Contact frequency | -0.001 | -0.000 | -0.001 |
|  | (0.001) | (0.000) | (0.001) |
| Constant | 0.440 | -0.448*** | 0.332** |
|  | (1.084) | (0.077) | (0.156) |
| R-squared | 0.111 | 0.128 | 0.095 |
| Control Variables | YES | YES | YES |
| Individuals FE | YES | YES | YES |
| Year FE | YES | YES | YES |

*Note*: ① *** p<0.01, ** p<0.05, † p<0.1. ② Robust standard errors clustered at the individual level are reported in parentheses.

Table A6. Heterogeneity Analysis in China

|  | FI | | | |
| --- | --- | --- | --- | --- |
| VARIABLES | Male | Female | One child | More than one child |
| Intergenerational care hours | 0.000 | -0.000 | 0.003 | -0.000 |
|  | (0.001) | (0.001) | (0.002) | (0.001) |
| Amount of downward intergenerational economic support | 0.001 | **0.002***** | 0.002† | 0.001*** |
|  | (0.001) | **(0.001)** | (0.001) | (0.000) |
| Amount of upward intergenerational economic support | 0.000 | 0.000 | -0.000 | 0.000 |
|  | (0.000) | (0.000) | (0.001) | (0.000) |
| Contact frequency | -0.001 | -0.002 | -0.001 | -0.001 |
|  | (0.001) | (0.001) | (0.002) | (0.001) |
| Constant | 1.561 | -1.670 | 0.827 | -0.094 |
|  | (1.844) | (1.372) | (2.499) | (1.336) |
| R-squared | 0.155 | 0.183 | 0.164 | 0.171 |
| Observations | 9,757 | 9,410 | 1,796 | 17,371 |
| Control Variables | YES | YES | YES | YES |
| Individuals FE | YES | YES | YES | YES |
| Year FE | YES | YES | YES | YES |

*Note*: ① *** p<0.01, ** p<0.05, † p<0.1. Bold denotes significance after Bonferroni correction (p < 0.00625). ② Robust standard errors clustered at the individual level are reported in parentheses.

Table A7. Heterogeneity Analysis in Korea

|  | FI | | | |
| --- | --- | --- | --- | --- |
| VARIABLES | Male | Female | One child | More than one child |
|  |  |  |  |  |
| Intergenerational care hours | -0.000 | -0.001 | 0.000 | -0.001 |
|  | (0.002) | (0.001) | (0.003) | (0.001) |
| Amount of downward intergenerational economic support | -0.001 | 0.003† | -0.006† | 0.001 |
|  | (0.001) | (0.001) | (0.003) | (0.001) |
| Amount of upward intergenerational economic support | -0.000 | **0.001***** | 0.002 | 0.001 |
|  | (0.001) | **(0.000)** | (0.001) | (0.000) |
| Contact frequency | -0.000 | 0.000 | 0.003 | 0.000 |
|  | (0.001) | (0.001) | (0.002) | (0.001) |
| Constant | -0.342*** | -0.309*** | -0.374*** | -0.275*** |
|  | (0.044) | (0.039) | (0.089) | (0.032) |
| R-squared | 0.069 | 0.083 | 0.113 | 0.075 |
| Observations | 8,984 | 12,039 | 1,245 | 19,778 |
| Control Variables | YES | YES | YES | YES |
| Individuals FE | YES | YES | YES | YES |
| Year FE | YES | YES | YES | YES |

*Note*: ① *** p<0.01, ** p<0.05, † p<0.1. Bold denotes significance after Bonferroni correction (p < 0.00625). ② Robust standard errors clustered at the individual level are reported in parentheses.

Table A8. Heterogeneity Analysis in the U.S.

|  | FI | | | |
| --- | --- | --- | --- | --- |
| VARIABLES | Male | Female | One child | More than one child |
| Intergenerational care hours | -0.001 | -0.001 | -0.001 | -0.001** |
|  | (0.001) | (0.001) | (0.002) | (0.001) |
| Downward intergenerational economic support | -0.000 | **0.004***** | 0.001 | 0.003** |
|  | (0.002) | **(0.001)** | (0.003) | (0.001) |
| Upward intergenerational economic support | **0.016***** | **0.016***** | **0.033***** | **0.014***** |
|  | **(0.004)** | **(0.003)** | **(0.007)** | **(0.003)** |
| Contact frequency | -0.000 | 0.000 | -0.000 | 0.000 |
|  | (0.000) | (0.000) | (0.000) | (0.000) |
| Constant | -0.232† | -0.472*** | -0.410† | -0.375*** |
|  | (0.127) | (0.115) | (0.231) | (0.092) |
| R-squared | 0.151 | 0.144 | 0.148 | 0.147 |
| Observations | 19,113 | 26,808 | 5,086 | 40,835 |
| Control Variables | YES | YES | YES | YES |
| Individuals FE | YES | YES | YES | YES |
| Year FE | YES | YES | YES | YES |

*Note*: ① *** p<0.01, ** p<0.05, † p<0.1. Bold denotes significance after Bonferroni correction (p < 0.00625). ② Robust standard errors clustered at the individual level are reported in parentheses.

Table A9. Heterogeneity Analysis in the U.K.

|  | FI | | | |
| --- | --- | --- | --- | --- |
| VARIABLES | Male | Female | One child | More than one child |
| Intergenerational care hours | 0.000 | 0.001 | 0.003 | 0.001 |
|  | (0.003) | (0.001) | (0.003) | (0.002) |
| Downward intergenerational economic support | 0.006*** | 0.003 | 0.005 | 0.004** |
|  | (0.002) | (0.002) | (0.004) | (0.002) |
| Contact frequency | 0.000 | -0.002 | -0.003 | -0.000 |
|  | (0.002) | (0.002) | (0.002) | (0.001) |
| Constant | 0.578† | 0.382† | -0.236 | 0.649*** |
|  | (0.298) | (0.198) | (0.371) | (0.201) |
| R-squared | 0.090 | 0.076 | 0.084 | 0.082 |
| Observations | 6,159 | 7,318 | 2,520 | 10,957 |
| Control Variables | YES | YES | YES | YES |
| Individuals FE | YES | YES | YES | YES |
| Year FE | YES | YES | YES | YES |

*Note*: ① *** p<0.01, ** p<0.05, † p<0.1. Bold denotes significance after Bonferroni correction (p < 0.00625). ② Robust standard errors clustered at the individual level are reported in parentheses.
